# Supplementary material for: The Gut Bacteria Dysbiosis Contributes to Chronic Graft-Versus-Host Disease Associated With a Treg/Th1 Ratio Imbalance
Source: Front Microbiol. 2022 Sep 8;13:813576. doi: 10.3389/fmicb.2022.813576 (PMC9493085; doi:10.3389/fmicb.2022.813576)
Supplement: Supplementary file 1 [file Data_Sheet_1.zip › P101SC18090073-01-B1-3-4_result/04.BetaDiversity/PCA/PCA12.pdf]

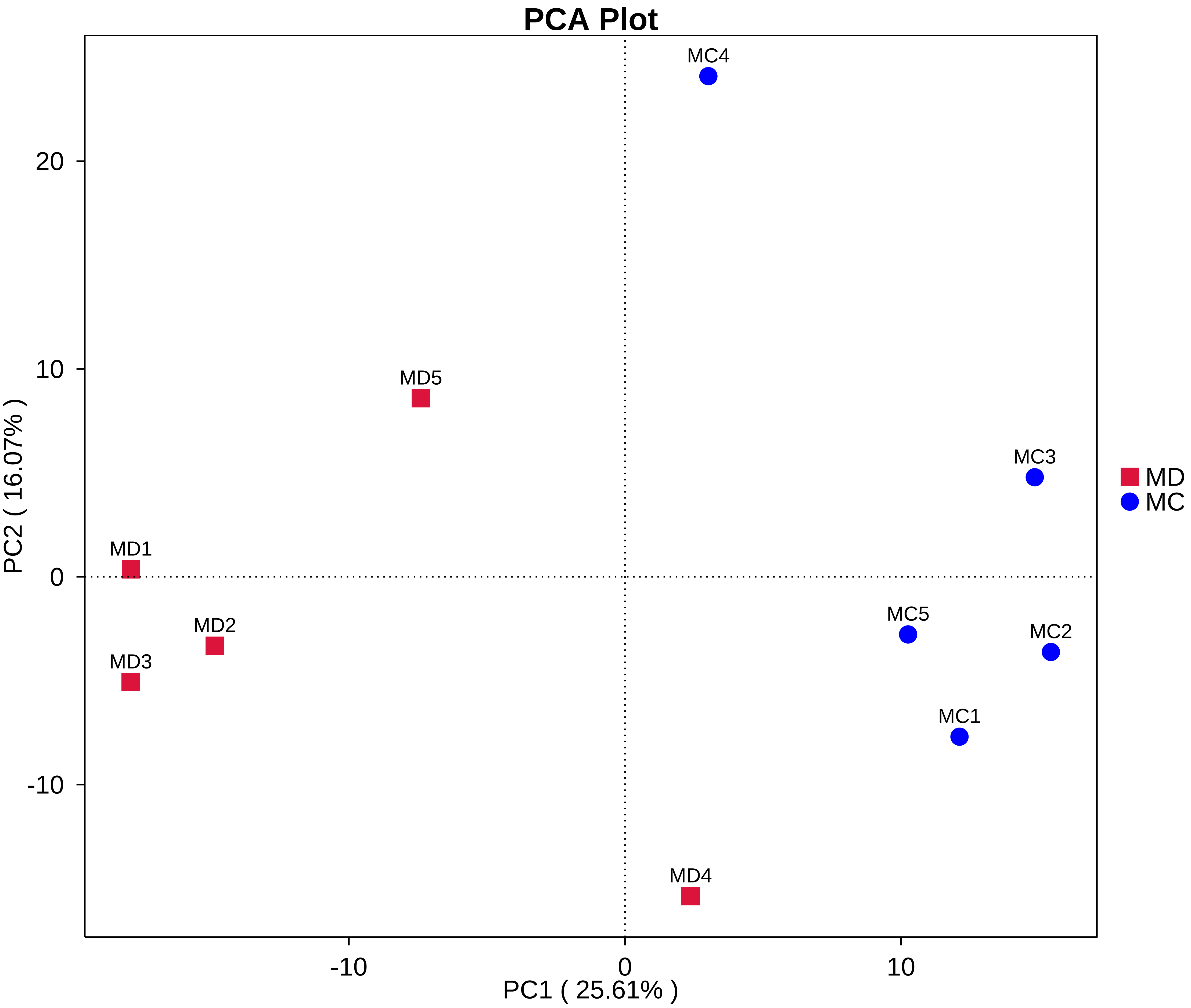
PCA Plot
MC4
20
10
MD5
MC3
MD
MC
PC2 (16.07%)
MD1
0
MD2
MC5
MC2
MD3
MC1
-10
MD4
-10
0
10
PC1 (25.61%)
